# Treatment of hospital wastewater by anodic oxidation using a new approach made by combining rotation with pulsed electric current on Cu-SnO_2_–Sb_2_O_5_ rotating cylinder anode

**DOI:** 10.1016/j.heliyon.2025.e42069

**Published:** 2025-01-16

**Authors:** Falah H. Abd, Ali H. Abbar

**Affiliations:** Department of Biochemical Engineering, Al-Khwarizmi College of Engineering, University of Baghdad, Baghdad, 10071, Iraq

**Keywords:** Tin oxide, Rotating cylinder electrode, Hospital wastewater, Anodic oxidation, Pulsed current

## Abstract

A high-efficiency, low-cost Cu-SnO_2_-Sb_2_O_5_ anode was prepared using a novel approach that combines the effects of rotation with pulsed current. The effects of operating variables such as rotation speed (50–250 rpm), pulsed current density (5–20 mA/cm^2^), and electrodepostion time (30–60 min) on the morphology and activity of Cu-SnO_2_–Sb_2_O_5_ anode were investigated. The structure of Cu-SnO_2_–Sb_2_O_5_ anode was examined by SEM, EDS, and XRD techniques. The results showed that using higher rotation speed combined with pulsed current gave better properties of Cu-SnO_2_–Sb_2_O_5_ anode in terms of higher oxidation activity and longer service life time. The optimum conditions for preparing Cu-SnO_2_–Sb_2_O_5_ anode were a pulsed current density of 10 mA/cm^2^, rotation speed of 250 rpm, and deposition time of 60 min. The prepared anode has the ability to remove methylene blue (MB) with an efficiency of 99.7 %. It has an excellent service life of 30 h. Additionally, the prepared anode has the potential to remove COD from hospital wastewater with 85 % efficiency by applying a current density of 10 mA/cm^2^ for 120 min at an initial pH of 3 where an energy consumption of 2.85 kWh/kg was claimed. The novel approach of combining rotation with pulsed electric current in preparing Cu-SnO_2_-Sb_2_O_5_ anode offers enhanced methylene blue degradation efficiency and extended anode life, demonstrating potential for industrial-scale hospital wastewater treatment.

## 1 Introduction

Hospitals need a large amount of fresh water to perform numerous tasks including medical facilities, hygienic practices, and others [1]. It was reported that the daily consumption of water per patient bed ranged between 0.1 m^3^ to 1.2 m^3^ depending on beds number at the hospital [2,3]. Therefore, huge amounts of hospital wastewater (HWW) are discharged and should be treated with a suitable technology before releasing them to the natural ecosystem [2]. HWW is composed of numerous emerging pollutants including pharmaceutically active compounds, antibiotic resistance genes, bacteria, heavy metals, and detergents [1,4]. Generally, HWW has high levels of total dissolved solids, chemical oxygen demand (COD), biochemical oxygen demand (BOD), nitrates, phosphates, and ammonia [5].

Various conventional methods were used to treat HWW comprising biological, chemical and physical processes. Among them, adsorption [6], nanofiltration [7], membrane bioreactors [8], and biological degradation [9] have been successfully used. However, these methods have many limitations, for example, adsorption process has low efficiency with generation huge amount of sludge as secondary pollutants while the biological method requires excessive energy consumption and labor intensive to grow the microbes used as well as has low efficiency with rapid saturation and bad odor [10]. Furthermore, HWW has biodegradability index (BOD to COD ratio) lower than the municipal wastewater which makes it difficult to treat by the traditional biological systems due to killing the microorganisms by pharmaceutical complex compounds [11,12]. Therefore, adopting an effective method with high removal rate of pollutants and less environmental impacts will be urgent.

Advanced technologies such as membrane technologies and advanced oxidation processes (AOPs) may be the promised substituted approaches instead of the biological methods for treating HWW [13]. AOPs involve formation of OH• radical as a none-selective oxidizing agent to attack all the organic pollutants in the HWW [14]. AOPs have been applied successfully for treating HWW [[15], [16], [17]]. Electrochemical advanced oxidation processes as an efficient group within AOPs have been recently paid attention in the treatment of HWW [[18], [19], [20], [21]]. Electrochemical advanced oxidation processes have high efficiency in the oxidation reactions, ecofriendly, easy for controlling, possess low energy consumption, and considerably low-cost processes [22].

Electrochemical oxidation (EO) or anodic oxidation (AO) is one of the electrochemical advanced oxidation processes which has received attractive attention recently for application in treatment of HWW due to the low energy consumption, ease of operation, fast reaction rate, and environmental compatibility [18]. However, the industrial application of EO is restricted due to the short life time, high cost, and sometimes lower current efficiency of anodes [23]. From a practical vie point, anodic materials should have high oxygen evolution, good conductivity and stability with superior electrochemical activity [24]. Accordingly, three anodic martials have been applied successfully namely: lead dioxide (PbO_2_) [25], boron-doped diamond (BDD) [26] and antimony –doped tin oxide (Sb-SnO_2_) [27] as a results of their high ability to generate OH• and their non-active surfaces. BDD is costly (15,000–2200$ per m^2^) and its preparation conditions are difficult that restrict its industrial application [28]. Additionally, tantalum and niobium that used as a substrate for BDD are very expensive [29]. Many works showed that BDD anodes possess larger resistance in comparison with PbO_2_ and SnO_2_ anodes resulting in higher cell voltage during the oxidation process [30]. Lead dioxide anode severed from heavy metal pollution issue hence its using in wastewater treatment is not preferable [31]. In contrast, Sb-SnO_2_ anode is cheap, easy to fabricated, and possess high catalytic activity [32]. Therefore, its application in treating HWW was found to be efficient [14,[33], [34], [35], [36], [37]].

Different approaches have been used to fabricate Sb-SnO_2_ anodes for treating wastewater, including ultrasonic spray pyrolysis [38], self-assembly [39], thermal decomposition [40], sol-gel [41], dip coating [42], hydrothermal synthesis [43], and electrodeposition [32]. Among these approaches, electrodepostion is a facile, simple, low temperature, inexpensive, and high yield method that could be scale-up to an industrial scale [44]. Different substrates have been used over which thin film of SnO_2_ was deposited, including titanium [24,32,[44], [45], [46]] from citrate bath, copper [[47], [48], [49]] and graphite [50] from nitrate baths. Zhang et al. [46] found that electrodepositing Cu on Ti prior to electrodepostion of SnO_2_ resulted in enhancement the bonding force between SnO_2_ and Ti. Furthermore, Sun et al. [44] and Wu et al. [51]found that citrate bath gives better coating for SnO_2_. However, preparation of SnO_2_ by electrodepostion on a copper substrate from a citrate bath was not investigated previously. Therefore, the main aim of present work is to prepare Sb-SnO_2_ on a copper substrate as a low cost and cheap material in comparison with titanium substrate.

Rotating cylinder electrodes (RDEs) have received attractive attention recently in the application of electrodepostion [52]. These electrodes have good mass transfer properties due to the formation of turbulent flow conditions at moderate rotation speeds. Additionally these electrodes exhibit uniform current and potential distributions leading to formation uniform coatings on their surfaces during the electrodepostion process [53]. RCEs offer a significantly larger surface area making their scale-up to industrial applications more feasible [53]. Therefore, they are used for electroplating, metal recovery, and metal powder production by cathodic deposition [[54], [55], [56], [57], [58], [59], [60]]. To the best of authors knowledge, application of rotating cylinder electrode for electrodeposition tin oxide was not reported previously.

During the electrodepostion of tin oxide two approaches have been applied: direct and pulsed electric current. Pulsed electrodeposition approach gives harder, more uniform, and pore free Nano sized coating in comparison with the direct current deposition [61]. Previous studies confirmed that tin oxide prepared by a pulsed current is thicker than that prepared by continues current and has high efficiency in the oxidation process [62]. Various works have been conducted for preparing tin oxide anode by a pulsed electrodepostion using a duty cycle with pulse frequency [62,63], anodic pulse, cathodic pulse with relaxation [46,51], and cathodic square-wave pulse [64].

In the present work, a new approach based on a combination of rotation with pulsed electric current during the cathodic preparation of Cu-SnO_2_–Sb_2_O_5_ anode was adopted. This approach has not been investigated previously. Adopting this new approach has many benefits; firstly combining rotation with pulsed current might be given a new compact with uniform deposition due to the effect of rotation and pulsing simultaneously. Secondly, the proposed approach is cost-effective and easy to scale up for an industrial stage without requiring to the more expensive pulse generating systems such as potentiostat or pulse rectifier. The effect of rotation speed, current density, and time on the morphology of SnO_2_ deposits have been investigated combining with examine the accelerated life time of the prepared anode. Application of the prepared anode for treating hospital wastewater has been examined via exploring the effect of current density and pH on the removal of COD as curtail parameters.

## 2 Experimental work

### 2.1 Materials and reagents

Methylene blue (MB) with a purity of 97 % was obtained from BDH Chemical Ltd Poole England. Gelatin was obtained from MP Biomedicals and used in all anode preparation experiments at a concentration of 0.8 g/L [46]. Citric acid was obtained from the laboratory reagent. SnCl_2._2H_2_O and SbCl_3_ were obtained from sisco research laboratories. Na_2_SO_4_ has a purity of 99 % and was purchased via the central drug house. Sodium hydroxide pellets (98 %) and sulfuric acid (98 %) were purchased from the laboratory reagent and used to adjust the pH of solution. A digital pH meter type (PH211, HNNA Instrument Inc., Romania) was used to measure the pH of solution.

No further purification was performed to all chemical compounds and used as received from the source. To prepare solutions for the experiments of anode preparation and oxidizing ability testing, deionized water was used.

Regarding to wastewater treatment, a sample of wastewater (5L) was taken from the collecting tank at the hospital of Al-Diwaniyah in Iraq. The wastewater has the following properties: pH = 7.8, COD = 590 mg/L, BOD = 130 mg/L, total dissolved solid = 2160 ppm, Cl^−^ = 0.6 g/L, SO_4_^2−^ = 0.4 g/L, turbidity = 21.2 (NTU), and conductivity = 2.29 mS/cm. The sample was kept at 4 °C during the experiments to insure no change in its properties.

### 2.2 Preparation of Cu-SnO_2_–Sb_2_O_5_ anode

**Fig. 1:**
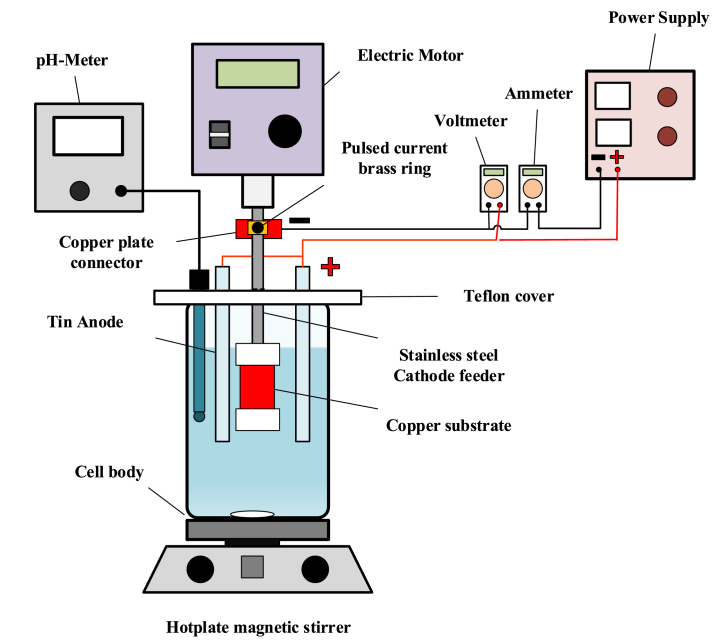
Electrodeposition system.

Fig. 1 represents the laboratory system used in preparing Cu-SnO_2_–Sb_2_O_5_ anode. It was composed from an electrochemical cell, hot plate magnetic stirrer (ISOLAB Laborgeräte GmbH, Germany), electric motor type (Global Lab, Republic of Korea) with rotation speed range from 0 to 1500 rpm, power supply type (UNI-T:UTP3315TF-L), Ammeter and voltmeter (Kwun Tong, Kowloon, Hong Kong), and pH meter. The electrochemical cell is composed of a cell body with its cover. The cell body is Pyrex beaker of 300 ml having dimensions (inside diameter of 7 cm and length of 11.5 cm). The cover is a ring type made from Teflon having inside diameter of 3 cm, outer diameter of 7 cm, and thickness of 2 cm. It was provided with four rectangular holes (1.5 × 0.7 cm) distributed equally on its surface. The external diameter of cover was reduced in sized by a distance of 10 mm for fixing the cover on the cell body. Four strips of tin were used as anode with a purity of 99 %. They are purchased from (PT Energi delapan mineral) and each one of them has dimensions (11 cm length, 1.5 width, and 0.7 cm thickness). They were screwed from the top blow the head by a distance of 10 mm to hold them via copper screws (5 mm in diameter and 20 mm in length) with nets. All strips were connected electrically.

The cathode was composed from a stainless steel feeder and a hollow copper cylinder used as a substrate for deposition of tin. It has a length of 10 mm, an outside diameter of 18 mm, and a thickness of 0.5 mm. The cathode feeder is a solid rod with length of 115 mm. It was composed from two sections: the lower has diameter of 17 mm and length of 30 mm, while the upper section has 8 mm diameter and 85 mm length. The lower section has two Teflon sleeves; the lower sleeve, with a closed-end, has dimensions (outside diameter of 20 mm, inside diameter of 16 mm, and thickness of 10 mm) while the upper sleeve, with an open-end, has dimensions (20 mm outside diameter, 16 mm inside diameter, and 15 mm thickness). Between these sleeves the copper cylinder was fixed and silicon rubber was inserted between the sleeves and copper cylinder to prevent the solution from attacking the current feeder. At the upper section of the cathode feeder, there is a brass ring with dimensions (outside diameter of 20 mm, inside diameter of 8.2 mm, and thickness of 10 mm) was fixed at a distance of from the upper end. At the lateral surface of brass ring there is screwed Teflon cap with diameter of 10 mm utilized to make on/off current (pulse action). The current was provided to the cathode feeder via a strip of copper attached to the brass ring and fixed on the stand of the electric motor.

Before start any run the copper cylinder was polished with sandpapers of 400 and 800 grits then cleaned via soaking in ethanol for a period of 15min followed by acetone for a period of 15min and finally in 0.1 M HCl with sonification using ultrasonic bath (ISOLAB Laborgeräte GmbH, Germany) for a period of 15 min. After that the copper cylinder was fixed on the cathode feeder.

The electrodepostion bath was composed from 67.5 g/L, SnCl_2_.2H_2_O, 2.25 g/L SbCl_3_ and 57.5 g/L citric acid [44]. In the present work, am acidic chloride-based electrolyte is adopted for electrodepostion of tin because an acidic bath is preferred as Sn^2+^ ions are the most participated species in the charge transfer step while complex ions such as Sn(OH)_6_^2-^ participate in the cathode reaction in case of using an alkaline bath which complicates the tin electrodepostion process [65].

The electrodepostion is started by putting 150 ml of solution in the cell body and heating to 50 °C. After that the electric motor was set on the required rotation speed followed by applying the required current density for a certain period of time. At the end of the deposition, cathode was dismantled to separate the copper cylinder which is coated with tin then rinsed thoroughly with distilled water and dried at 100 °C for 1 h then calcined at 550 °C for a period of 3 h using muffle furnace model:MF-12/HYSC, Korea.

### 2.3 Characterization of the anode

The crystal structure and morphology of Cu/SnO_2_-Sb_2_O_5_ anode were examined via SEM type (FEI Company Netherlands) using the following operating conditions: HV = 25 kV, bias = 1400 V, bias = 0, and spot = 8.0. The crystal structure of the Cu/SnO_2_-Sb_2_O_5_ anode was identified by X-ray diffractometer type (XRD 6000/Shimadzu/Japan) with CuKα radiation of 1.5405 Å and testing range of 2θ between 20 and 80° with scan step time of 1.2 s and a step size of 0.2°. The XRD was operated at 40 kV and 30 mA.

### 2.4 Accelerated service life test

The accelerated service life test was conducted using the same system in section 2.2 but with using the prepared Cu-SnO_2_–Sb_2_O_5_ as an anode and a cathode made of hollow graphite cylinder (70 mm outside diameter and 80 mm length). The electrolyte was 0.5M NaOH, and a current of 200 mA was applied. The cell voltage was recorded with time and the anode was considered to be deactivated once the cell voltage increased sharply beyond 2 V with respect to the overall cell voltage [66]. Based on the experimental observation, utilizing H_2_SO_4_ as a test solution was excluded because H_2_SO_4_ attacked Cu substrate via grooves located between the sleeves and copper cylinder leaving SnO_2_ alone as a compact film. This phenomena was combined with continuous decreasing in the cell voltage as a result of Cu precipitation on the cathode.

### 2.5 Anode performance

The oxidizing ability of Cu-SnO_2_–Sb_2_O_5_ anode was estimated via anodic oxidation of methylene blue (MB). 100 ml of aqueous solution containing 100 mg/L MB with 0.25M sodium sulfate (supporting electrolyte) was used. A constant current density of 20 mA/cm^2^ was applied across the Cu-SnO_2_–Sb_2_O_5_ rotating cylinder anode and the hollow graphite cathode for 4 h under anodic rotation speed of 500 rpm. The choice of graphite as a cathode is based on previous studies [67]. Samples were taken every 1 h for determine the remaining concentration of MB by measuring its absorbance at a wavelength of 664 nm using UV–Vis spectrophotometer type (Shimadzu/Japan UV–Vis spectrophotometer) then color removal efficiency was estimated by Eq. (1) [44]:

(1) $$\text{Decolorization}\;\text{efficiency}\left(\text{RE}\%\right)=\frac{A_{o}-A_{t}}{A_{o}}\times 100$$

where A_o_ and A_t_ are the initial and final absorbance of MB at time (t).

Most of the direct anodic oxidations of MB on SnO_2_ anodes were occurred under mass transfer control which was subjected to a pseudo-first-order kinetic model according to Eq. (2) [44,68,69]:

(2) $$\ln\left(\frac{A_{o}}{A_{t}}\right)=K_{obs.}t$$

where (K_obs._) is the pseudo-first order rate constant.

### 2.6 Treatment of hospital wastewater

**Fig. 2:**
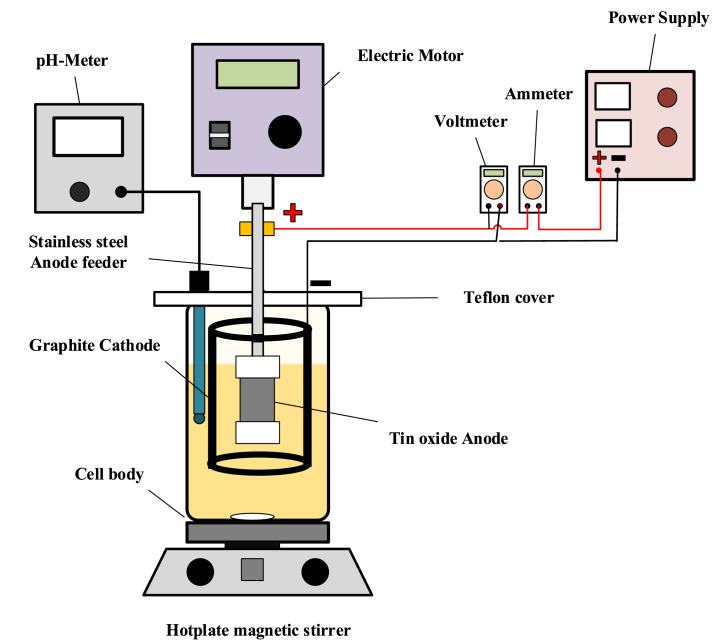
Electrochemical system for treating hospital wastewater.

The electrochemical system is shown in Fig. 2 which similar to that used in section 2.2 with the following exceptions: the anode is Cu-SnO_2_–Sb_2_O_5_ while the cathode is a hollow cylinder made of graphite with a length of 80 mm and outside diameter of 70 mm. The brass ring was replaced by copper ring without Teflon cap.

The anodic treatment was performed by taken 200 ml of the wastewater and adding 0.05 M Na_2_SO_4_ as a supporting electrolyte. The required current density was applied for a period of 120 min after adjusting the pH of solution at the suitable initial value. The anodic treatment was performed using a direct current at a rotation speed of 250 rpm.

At the end of treatment, a sample was taken for measuring the COD by digesting it with dichromate for 120 min at 150 °C using thermo reactor type (RD-125, Lovibond). COD is measured by photometer (MD 200 COD VARIO Photometer, Lovibond, Germany).

The COD elimination efficiency was estimated by Eq. (3) [20]:

(3) $$RE\%=\frac{C_{o}-C_{f}}{C_{o}}\times 100$$

where C_o_ and C_f_ represents the initial and final values of COD (mg/l).

The specific energy consumption (SEC) value represents the quantity of energy consumed during the process of digesting a kilogram of COD. SEC in (kWh/kgCOD) can be calculated using eq. (4) [20].:

(4) $$\mathrm{SEC}=\frac{E\times I\times t\times 1000}{\left(C_{o}-C_{f}\right)\times V}$$

E denotes the applied cell voltage (Volt), t denotes the electrolysis period (h), I denotes the current (A), V denotes the effluent volume (L).

For the sample of wastewater the initial and final values of BOD was determined by BOD-System BD600, Lovibond.

## 3 Results and discussion

### 3.1 Preparation of Cu/SnO_2_-Sb_2_O_5_ anode

#### 3.1.1 Direct current/Effect of rotation speed

**Fig. 3:**
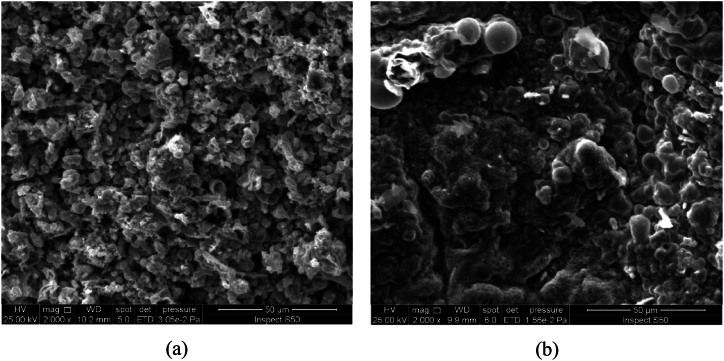
SEM images of Cu/SnO_2_-Sb_2_O_5_ anode prepared at a current density of 10 mA/cm^2^ (DC). a)50 rpm, b)250 rpm.

**Fig. 4:**
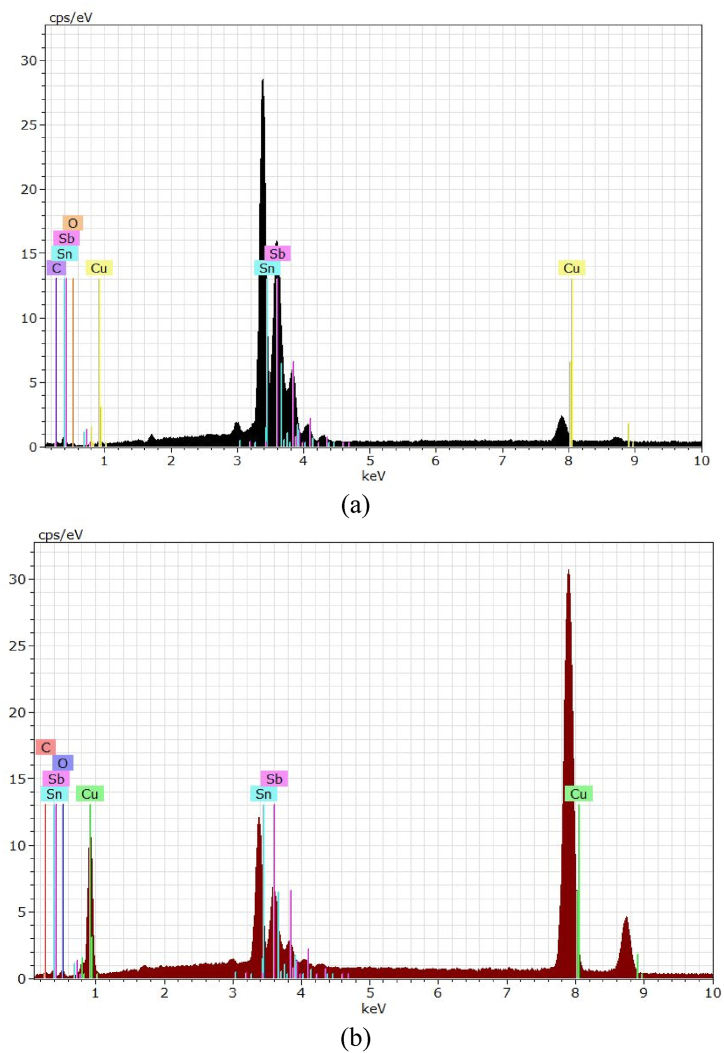
EDS for Cu/SnO_2_-Sb_2_O_5_ anode prepared at a current density of 10 mA/cm^2^ (DC). a)50 rpm, b)250 rpm.

**Fig. 5:**
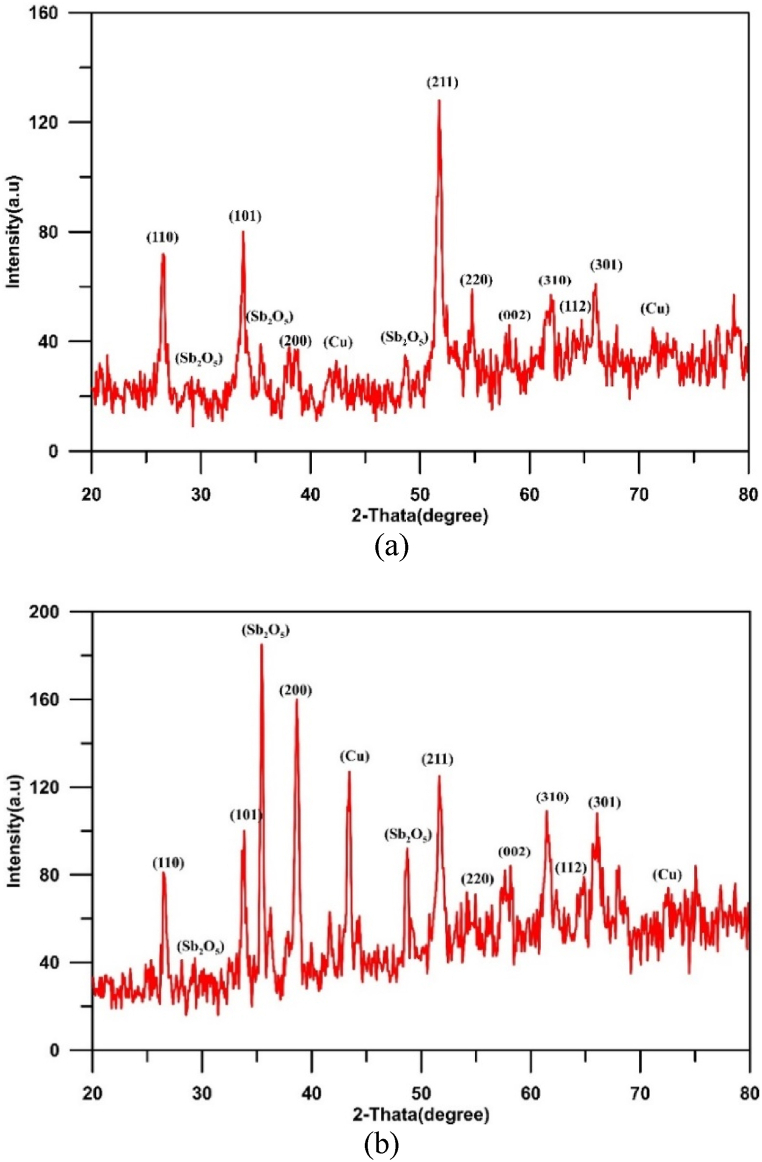
XRD results for Cu/SnO_2_-Sb_2_O_5_ anode prepared at a current density of 10 mA/cm^2^ (DC). a)50 rpm, b)250 rpm.

The effect of rotation speed under direct current supplying on the morphology and the structure of deposited tin oxide has been investigated at a constant current density of 10 mA/cm^2^ for a period of 30 min. Fig. 3(a and b) and Fig. 4(a and b) show SEM and EDS results while Fig. 5(a and b) represents the XRD results.

Fig. 3(a and b) shows the SEM images of Cu/SnO_2_-Sb_2_O_5_ anodes prepared by cathodic deposition at 50 rpm and 250 rpm. The morphology in both rotation speeds were multilayer coating deposits with no crakes due to the using of citric acid which formed a stannous citrate complex on the surface of the film [32,44]. It can be observed that increasing the rotation speed results in a smaller particle size with more compact and uniform surface. This can be interpreted as increasing the rotation speed would reduce the boundary layer effect near the cathode causing more Sn^2+^ ions to be transported near the cathode surface and achieving high deposition rate with uniform deposition which consequently results in compact deposits having small particle size hence leading to increase the internal surface area and active sites on the anode surface [70].

Kazimierczak et al. [70] found that increasing the rotation speed results in a reduction in the particle size of the coatings that generated by deposition of Sn, Zn, Bi on a copper substrate. Wu et al., 2021 [60] found that the roughness of surface decreases with increasing rotation speed during the cathodic deposition of CoMnP films on copper. Similar behavior regarding the roughness of film generated in preparing a copper/tin alloy by electrodepostion [71].

Fig. 4(a and b) shows the EDS results where Sn, Sb, and O are detected confirming the formation of mixture oxides in the anode at two rotation speeds (50 and 250 rpm). The peak of Cu was clearly observed which related to the substrate of the anode. In addition, the peak at 8.75 keV belongs to rhenium which comes from the copper substrate. It is known that rhenium is mainly present as a by-product during the extraction of copper ores.

The quantitative analysis of EDS (atomic percentage) were 56.41%O, 30.45%Sn, 5.74 % Sb, 6.04%Cu, 0.65%Re, 0.71 % C for Cu/SnO_2_-Sb_2_O_5_ anode prepared at a current density of 10 mA/cm^2^ (DC) and 50 rpm. Regarding to Cu/SnO_2_-Sb_2_O_5_ anode prepared at a current density of 10 mA/cm^2^ (DC) and 250 rpm, the quantitative analysis of EDS were 58.41%O, 26.13%Sn, 5.9 % Sb, 6.83%Cu, 1.95%Re, 0.78 % C.

Fig. 5(a and b) displays the XRD results for Cu/SnO_2_-Sb_2_O_5_ anodes prepared by cathodic deposition at 50 rpm and 250 rpm. For both cases, the strong diffractions peaks of tetragonal rutile SnO_2_ were appeared at 2θ = 26.6°(110), 33.9°(101), 37.9°(200), 51.5°(211), 54.8°(220), 57.8°(002),61.9°(310), 64.7°(112), and 65.9°(301) which is corresponding to (PDF no. 41–1445).

According to literature, the crystal orientation depends on the physical structure of SnO_2_ and the growth rate during the preparation by deposition. In the case of SnO_2_ powder, the reflection that shows the strongest intensity of all peaks is the (110) plane, while in the case of SnO_2_ thin film, (211) or (200) planes have the highest [72]. Additionally, the preferred orientation of crystal plane depends on the surface energy of interaction between the film and substrate; the orientation of plane with lower surface energy of interaction is favored [73].

For 50 rpm, the strong peak is at (211) plane approving the preferred orientation towards (211) crystallographic direction, while in case of 250 rpm, the strong peak is at (200) plane approving the preferred orientation towards (200) crystallographic direction [44,74].

The strong texture along the (200) orientation has been reported to correspond to the best balance between the electrical resistivity and optical transmittance [75]. Additionally, Miao et al. [76] found that (200) plane has a minimum surface energy of interaction between the film and substrate. In all cases, peaks of copper were observed at 43.3° and 74.7° [48]. Peaks of Sb_2_O_5_ were observed at 2θ = 28.4°, 30.1°, 35.2°and 48.7° corresponding to the cubic crystallographic phase (COD1010505) [77]. No peaks for Sn was observed confirming that all tin convert to its oxide [44]. The crystal size of SnO_2_ was estimated by the Debye-Scherrer equation to be 55.25 nm at 50 rpm and 41.3 nm at 250 rpm.

Results showed that half peak widths at (110), (101), and (211) peaks increase as the rotation speed increases. According to Scherer's equation, the half peak width is proportional inversely with size of crystal [41], therefor increasing rotation speed results in obtaining smaller crystal size hence increasing active sites with the structure of anode. Similar observation was noted by Duan et al. [41], .

**Fig. 6:**
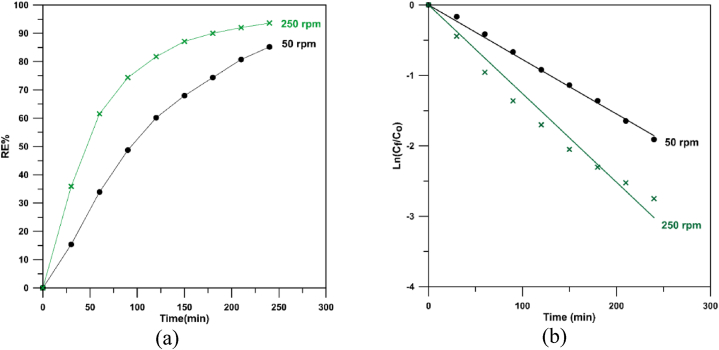
Decolorization of methylene blue at 20 mA/cm^2^ for 50 and 250 rpm (DC). a) Removal efficiency, b) Ln (C_f_/C_o_) versus time.

Fig. 6-a displays MB degradation by anodic oxidation utilizing different Cu/SnO_2_-Sb_2_O_5_ anodes at two rotation speeds (50 rpm and 250 rpm) where the results confirm a good catalytic activity of the anode prepared under 250 rpm where removal efficiencies of 61.54 and 93.6 % were achieved at 1 and 4 h respectively. The higher activity of Cu/SnO_2_-Sb_2_O_5_ anode prepared at 250 rpm may be due to the higher internal surface area and generation of more active sites for MB degradation as revealed by the SEM image which shows more homogeneity in the deposits with smaller particle size compared to the operating conditions at 50 rpm.

Fig. 6-b displays the plot of ln (C_f_/C_o_) verses time for Cu/SnO_2_-Sb_2_O_5_ anodes prepared at 50 and 250 rpm as rotation speeds. All fitting lines have R^2^ between 0.999 and 0.991 confirming the oxidation obeyed a pseudo first order behavior. From Fig. 6-b, the rate constant at 250 rpm was found to be 0.01258 min^−1^ which is higher than that at 50 rpm (0.00774 min^−1^) by 1.6 confirming the good performance and activity of Cu/SnO_2_-Sb_2_O_5_ anodes prepared at 250 rpm. Additionally, the rate constant of the present work is twice that manufactured by the sol-gel method (0.006 min^−1^) [41] approving a good catalytic activity of Cu/SnO_2_-Sb_2_O_5_ prepared by cathodic deposition.

#### 3.1.2 Pulsed electrodepostion

**Fig. 7:**
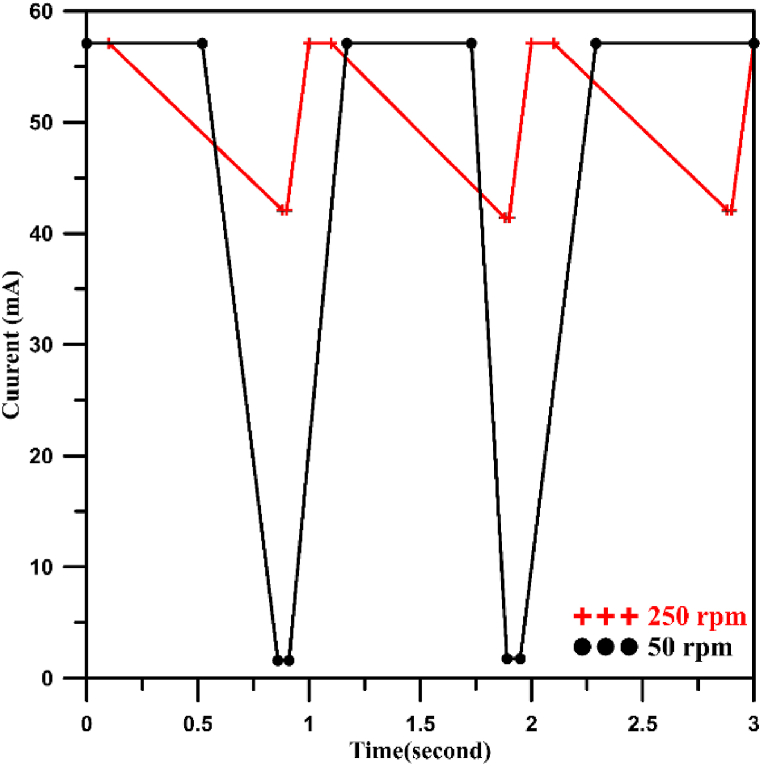
Pulse current diagram at 50 and 250 rpm.

A simple method was adopted to determine the shape of pulsed current at 50 and 250 rpm as shown in Fig. 7 by recording the variation of current at the power supply with time during the experiment using a high precision digital camera. In case of 250 rpm, the current varied between 57 and 42 mA for a cycle period of 1.0 s while for 50 rpm the current varied between 57 and 1.6 mA for a cycle period of 1.17 s. For a period of 3s, no. of pulses was 3 for 250 rpm while it was 2 for 50 rpm.

##### 3.1.2.1 Effect of rotation speed

**Fig. 8:**
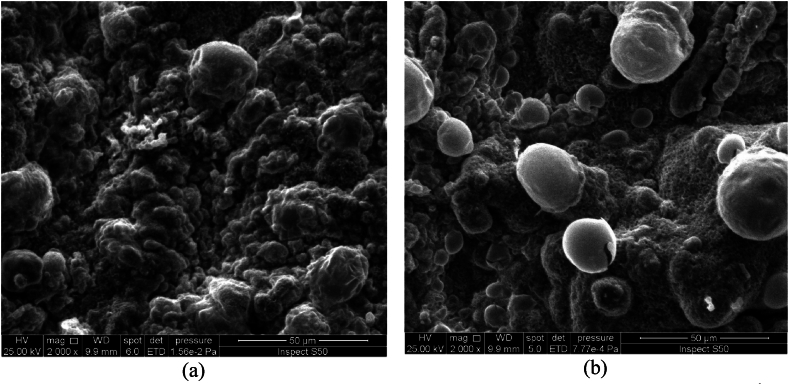
SEM images of Cu/SnO_2_-Sb_2_O_5_ anode prepared at a current density of 10 mA/cm^2^ (pulse). a)50 rpm, b)250 rpm.

**Fig. 9:**
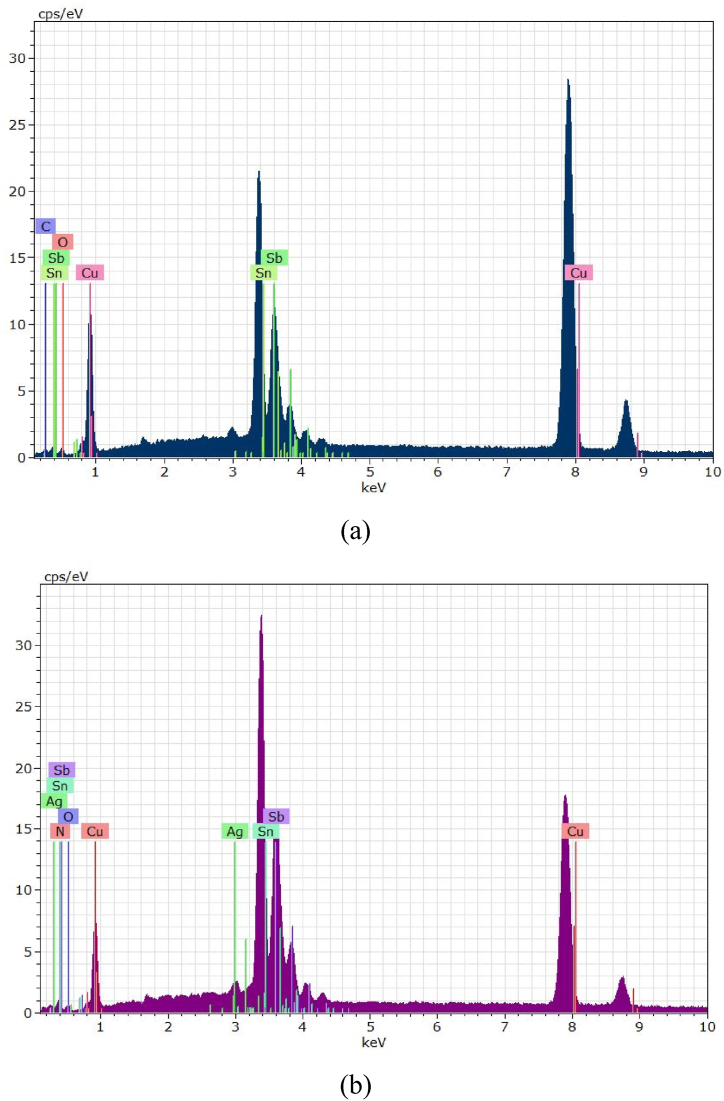
EDS for Cu/SnO_2_-Sb_2_O_5_ anode prepared at a current density of 10 mA/cm^2^ (pulse). a)50 rpm, b)250 rpm.

**Fig. 10:**
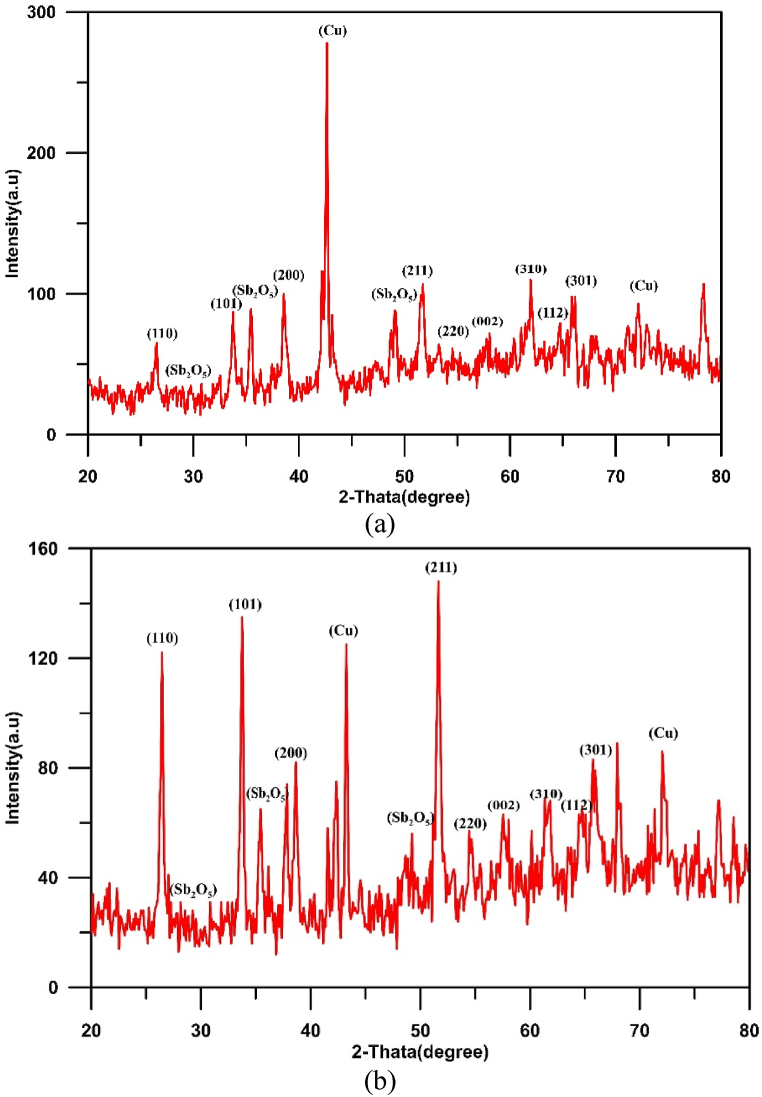
XRD results for Cu/SnO_2_-Sb_2_O_5_ anode prepared at a current density of 10 mA/cm^2^ (pulse). a)50 rpm, b)250 rpm.

The effect of rotation speed combined with pulsed current on the morphology and structure of deposited tin oxide has been investigated at constant current density of 10 mA/cm^2^ for a period of 30 min. Fig. 8(a and b) and Fig. 9(a and b) show the SEM and EDS results while Fig. 10(a and b) represents the XRD results. Fig. 8(a and b) illustrates that high speed with pulsed current results in a film structure with smaller particle size. Additionally the shape of particles seems to be spherical with a large proportion in comparison with deposits at 50 rpm. The reason for this configuration is the effect of pulsed current where increase the pulse number reduces the concentration polarization during the electrodepostion in which the nucleation process is accelerated while the growth rate is reduced leading to variation in the crystal growth direction to form small particle size [24,78].

Duan et al., 2016 [24] found similar morphology in fabricatiing Ti/Sb-SnO_2_ by pulsed electrodepostion. Semispherical deposits were observed by Zhang et al. [46] in preparing Ti/Sb-SnO_2_ modified with carbon nanotube using pulsed current technique. Hessam et al. [62] observed flower –like deposits in preparing SnO_2_ on a graphite substrate from HNO_3_ media by pulsed electrodeposition confirming that the type of electrolyte has an effect on the structure of deposits. Fig. 9(a and b) displays the EDS results in which peaks of Sn, Sb, and O were detected at two rotation speeds under pulsed current approving formation of SnO_2_ and Sb_2_O_5_. Similarly, peak of Cu was detected which relates to the substrate of anode.

Regarding to Cu/SnO_2_-Sb_2_O_5_ anode prepared at a current density of 10 mA/cm^2^ (pulse) and 50 rpm, the quantitative analysis of EDS (atom percentage) were 59.34%O, 26.13%Sn, 6.14 % Sb,6.23%Cu, 1.45%Re, 0.71 % C. While for 250 rpm were 59.11%O, 26.15%Sn, 5.79 % Sb, 6.99%Cu, 0.95%Re, 0.67 % C, 0.21%Ag, 0.13 % N.

Fig. 10(a and b) shows the XRD results for Cu/SnO_2_-Sb_2_O_5_ anodes prepared by cathodic deposition at pulsed current 10 mA/cm^2^ with two rotation speeds (50 and 250). Many diffractions peaks of tetragonal rutile SnO_2_ were observed matching to (PDF no. 41–1445). The strong peak is at (211) plane approving the preferred orientation towards (211) crystallographic direction in both rotation speeds [44,74]. The crystal size of SnO_2_ was estimated by the Debye-Scherrer equation to be 43.85 nm at 50 rpm and 35.7 nm at 250 rpm.

**Fig. 11:**
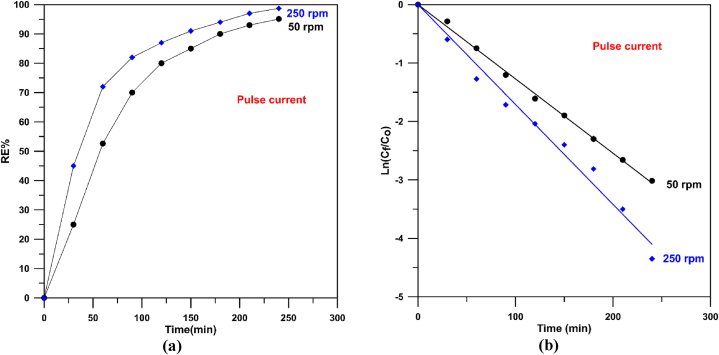
Decolorization of methylene blue at 50 and 250 rpm with pulsed current. a) removal efficiency, b) Ln (C_f_/C_o_) versus time.

Fig. 11-a displays MB degradation by anodic oxidation utilizing different Cu/SnO_2_-Sb_2_O_5_ anodes prepared at 10 mA/cm^2^ (pulsed current) and two rotation speeds (50 rpm and 250 rpm) where the results confirm a good catalytic activity of the anode prepared under 250 rpm (pulsed current) where removal efficiencies of 72 % and 98.71 % were achieved at 1 and 4 h respectively. While removal efficiency at 50 rpm not exceeded 95 % at 4 h. Generally, both rotation speeds under pulsed current give good activity in comparison with the direct current due to the effect of pulsing.

Fig. 11-b displays the plot of ln (C_f_/C_o_) verses time for Cu/SnO_2_-Sb_2_O_5_ anodes prepared at 10 mA/cm^2^ (pulsed current) and 50 an 250 rpm as rotation speeds. All fitting lines have R^2^ between 0.999 and 0.995 confirming the oxidation obeyed a pseudo first order behavior.

From Fig. 11-b, the rate constant at 250 rpm was found to be 0.0179 min^−1^ which is higher than that at 50 rpm (0.0127 min^−1^) by 1.4 confirming the good performance and activity of Cu/SnO_2_-Sb_2_O_5_ anodes prepared at 250 rpm. Furthermore, the rate constant at 250 rpm for pulsed current is 1.4 that in the direct current confirming the significant of pulsing effect. Additionally, the rate constant of present work is approximately three times of that made by sol-gel process (0.006 min^−1^) [41] approving a good catalytic activity for the Cu/SnO_2_-Sb_2_O_5_ prepared by cathodic deposition.

##### 3.1.2.2 Effect of current density

**Fig. 12:**
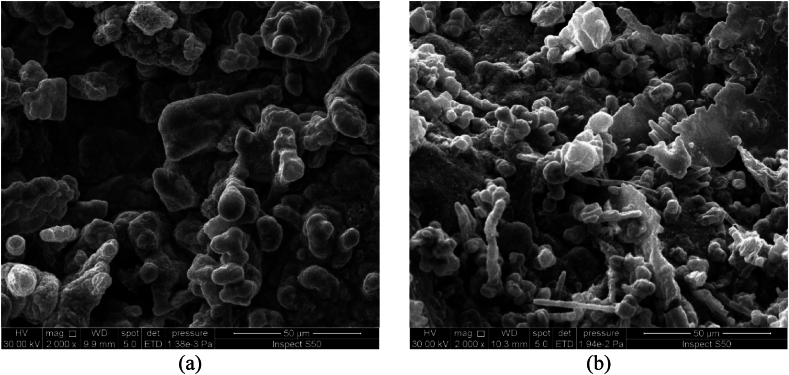
SEM images of Cu/SnO_2_-Sb_2_O_5_ anode prepared at 250 rpm. A) current density of 5 mA/cm^2^ (pulse), b) current density of 20 mA/cm^2^ (pulse).

**Fig. 13:**
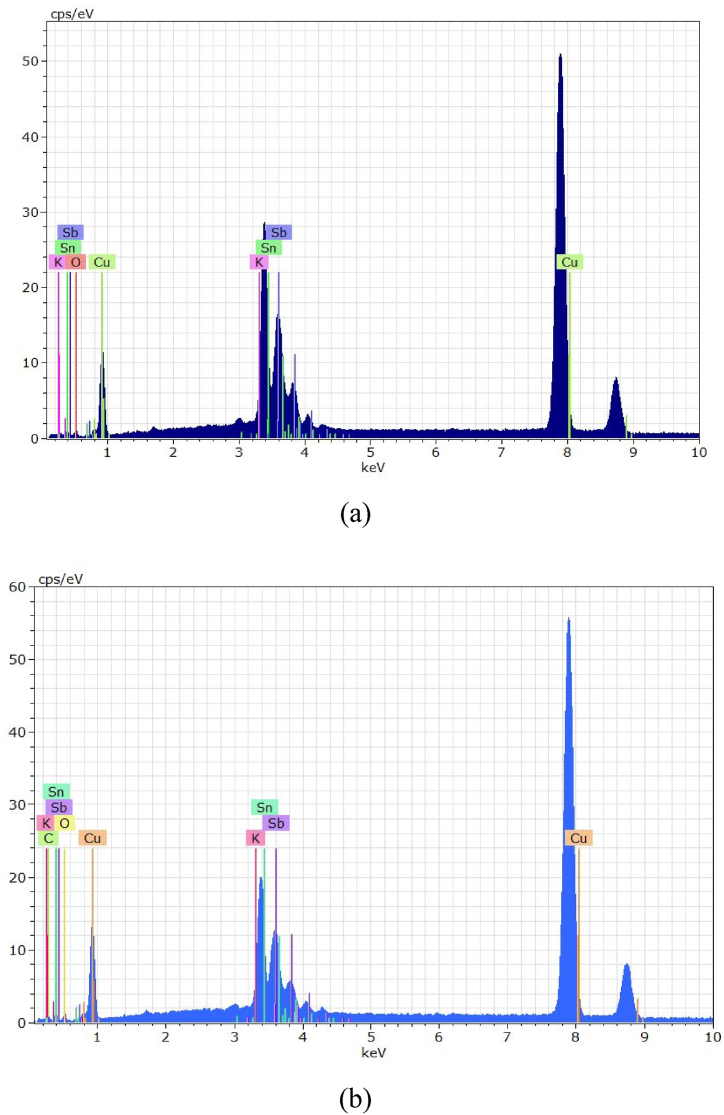
EDS for Cu/SnO_2_-Sb_2_O_5_ anode prepared at 250 rpm. A) Current density 5 mA/cm^2^ (pulse), b) current density 20 mA/cm^2^ (pulse).

**Fig. 14:**
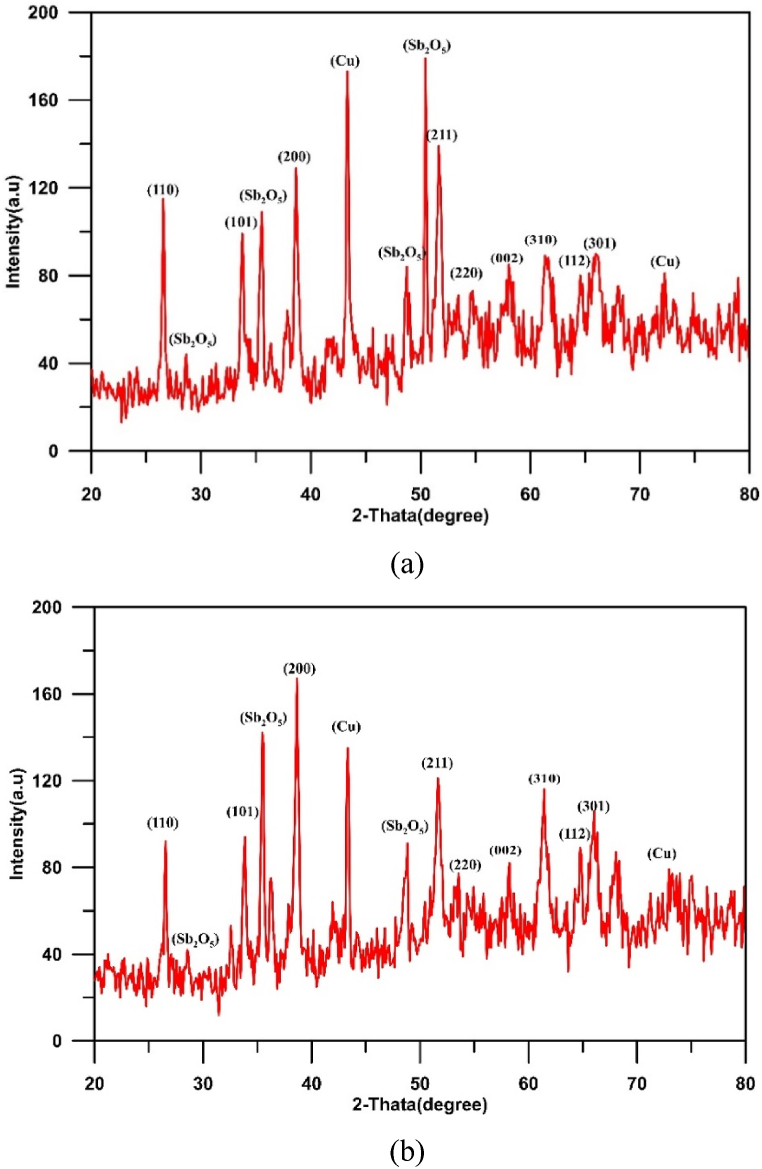
XRD results for Cu/SnO_2_-Sb_2_O_5_ anode prepared at 250 rpm. A) current density of 5 mA/cm^2^ (pulse), b) current density of 20 mA/cm^2^ (pulse).

The effect of current density under rotation combined with pulsed effect on the morphology and structure of the deposited tin oxide has been investigated at a rotation speed of 250 rpm for a period of 30 min. Fig. 12(a and b) and Fig. 13(a and b) show SEM and EDS results while Fig. 14(a and b) represents the XRD results. Fig. 12(a and b) displays the SEM images for two pulsed current densities (5 and 20 mA/cm^2^) where increasing the current density to 20 mA/cm^2^ gives deposits with smaller particle size and irregular shapes involving needle like configuration. Additionally, the structure seems to be more porous. These observations are in consistent with previous studies in which the increase in current density causes an increase in the nucleation rate leading to fine grain size [79]. Hessam et al. [62] observed that increasing the current density results in decreasing the crystallite size of film deposited. Furthermore, using high current density could be resulted in formation more porous deposits [62]. Sharma et al. [80] investigated thoroughly the effect of current density on the pulsed electrodepostion of tin and observed that increasing the current density results in increasing the rate of H_2_ evolution on the cathode leading to formation non-uniform porous structure of deposits. Fig. 13(a and b) shows EDS results in which also peaks of Sn, Sb, and O were observed which relates to SnO_2_ and Sb_2_O_5_

Regarding to Cu/SnO_2_-Sb_2_O_5_ anode prepared at 250 rpm and a current density of 5 mA/cm^2^ (pulse), the quantitative analysis of EDS (atom percentage) were 54.20%O, 26.14%Sn, 8.74 % Sb,8.24%Cu, 1.65%Re, 0.70 % C,0.33%K. While for 20 mA/cm^2^ were 59.71%O, 25.54%Sn, 7.24 % Sb,6.04%Cu, 0.65%Re, 0.71 % C,0.11%K. Fig. 14(a and b) shows the XRD results for Cu/SnO_2_-Sb_2_O_5_ anodes prepared at tow pulsed current densities 5 and 20 mA/cm^2^ under rotation speed of 250 rpm. Several diffractions peaks belong to tetragonal rutile SnO_2_ were observed which correspond to (PDF no. 41–1445). The strong peak is at (211) plane approving the preferred orientation towards the (211) crystallographic direction for 5 mA/cm^2^. Same orientation regarding to 10 mA/cm^2^ but change to orientation along (200) in case of 20 mA/cm^2^ [44,74]. The peak of Sb_2_O_5_ at 35° is higher in case of 20 mA/cm^2^ confirming the high deposition amount of antimony oxide with increasing the current. The crystal size of SnO_2_ was estimated by the Debye-Scherrer equation to be 73.65 nm at 5 mA/cm^2^ and 31.7 nm at 20 mA/cm^2^.

**Fig. 15:**
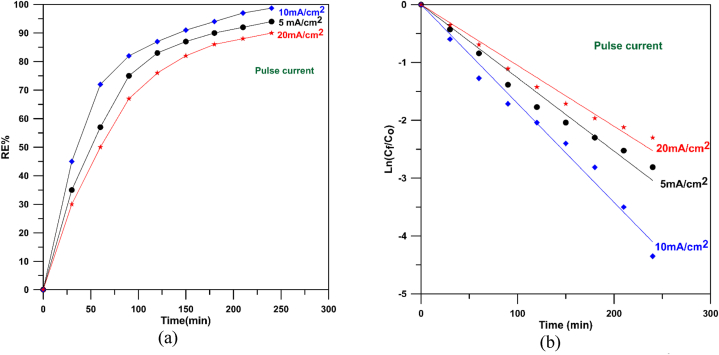
Decolorization of methylene blue at 250 rpm for current densities of 5–20 mA/cm^2^ (pulse). a) Removal efficiency, b) Ln (C_f_/C_o_) versus time.

Fig. 15-a displays MB degradation by anodic oxidation utlizing different Cu/SnO_2_-Sb_2_O_5_ anodes prepared at rotation speed of 250 rpm and different pulsed current densities where the results exhibited a good catalytic activity of the anode prepared under 10 mA/cm^2^ where removal efficiencies of 72 % and 98.71 % were achieved at 1 and 4 h respectively. While using higher current density results in reducing the removal efficiency to 90 %.

Fig. 15-b displays the plot of ln (C_f_/C_o_) verses time for Cu/SnO_2_-Sb_2_O_5_ anodes prepared at 250 rpm as rotation speed with different current densities. All fitting lines have R^2^ between 0.999 and 0.992 confirming the oxidation obeyed a pseudo first order behavior.

From Fig. 15-b, the rate constant at 10 mA/cm^2^ was found to be 0.0179 min^−1^ which is higher than that at 5 mA/cm^2^ (0.01266 min^−1^) by 1.4 and higher than that at 20 mA/cm^2^ (0.0105) by 1.7 confirming the good performance and activity of Cu/SnO_2_-Sb_2_O_5_ anodes prepared by cathodic deposition at 250 rpm and current density of 10 mA/cm^2^.

##### 3.1.2.3 Effect of time

**Fig. 16:**
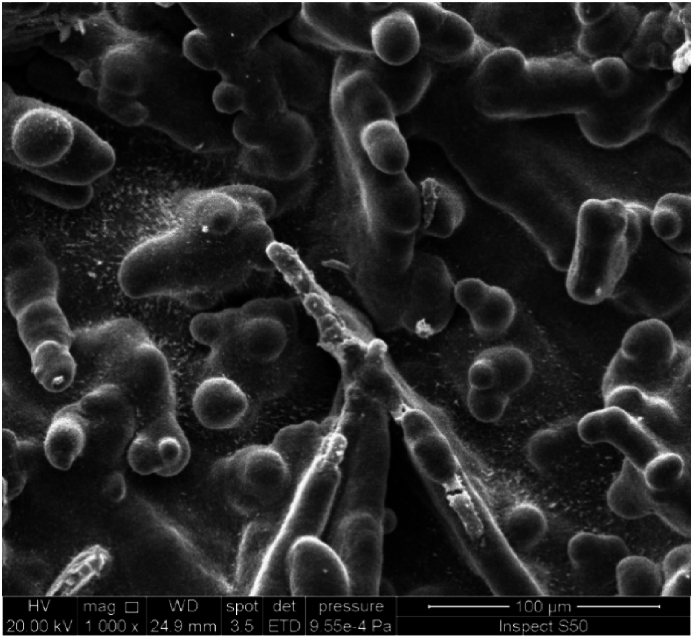
SEM images of Cu/SnO_2_-Sb_2_O_5_ anode prepared at 250 rpm and 10 mA/cm^2^ (pulse) and 60 min.

**Fig. 17:**
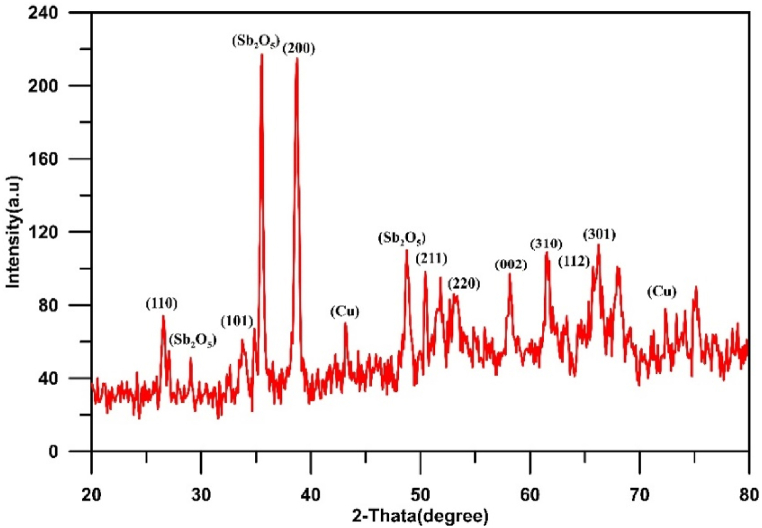
XRD results for of Cu/SnO_2_-Sb_2_O_5_ anode prepared at 250 rpm and 10 mA/cm^2^ (pulse) and 60 min.

The effect of time under rotation and pulsed effect on the morphology and structure of deposited tin oxide has been investigated at a rotation speed of 250 rpm and a current density of 10 mA/cm^2^. Fig. 16 shows the SEM results while Fig. 17 represents the XRD results.

Fig. 16 shows compact and none-porous structure of anode as the time of deposition increase to 60 min. This structure provides an improvement in the stability of anode and prevents the contact between the copper substrate and electrolyte causing long life of anode [24]. Zhao, et al., 2016 [81] observed the same structure regarding to the effect of time in preparation of Ti/SnO_2_-Sb_2_O_3_/PbO_2_ electrodes. No significant difference in EDS results was observed in comparison with that observed at 30 min.

It is important to compare the morphology of deposits by the present method with deposits prepared by other methods like sol-gel and thermal decomposition processes. Most of oxide electrodes prepared by the sol-gel or thermal decomposition have a cracked mud-like structure [82]. Previous studies on preparing SnO_2_ on a titanium substrate by sol-gel method showed that cracks formation would result in formation of titanium oxide hence affecting the stability of anodes [41].

Fig. 17 illustrates the XRD results for Cu/SnO_2_-Sb_2_O_5_ anodes prepared at pulsed current of 10 mA/cm^2^ and 60 min. Many diffractions peaks related to tetragonal rutile SnO_2_ were observed in this case which correspond to (PDF no. 41–1445). A strong peak was observed at (200) plane approving the preferred orientation towards (200) crystallographic direction at high deposition time [44,74]. Furthermore, Sb_2_O_5_ peaks are strong in case of high deposition time which confirm formation more Sb_2_O_5_within SnO_2_ deposits leading to increase the conductivity of anode. While peaks of Cu were shorter confirming of deposition thick film of tin oxide. Korotkov et al. [83], found that with increasing thickness of the SnO_2_ layers, the predominate orientation of SnO_2_ is altered to (200). The crystal size of SnO_2_ was estimated by the Debye-Scherrer equation to be 33.3 nm at 60 min.

As a final conclusion in comparison of XRD results with previous studies, it was observed that most of these works confirmed that SnO_2_ crystalline structure is based on peaks with crystalline orientation of (110), (101), and (211) 0r (200) as the preferred peaks in comparison with other peaks in the XRD. Furthermore most of previous works compared between the intensity of crystal orientations of (110), (101), (211) and/0r (200) and a further improvement in the electrode activity could be related to the strength of (211) 0r (200) which is in agreement with our results [65].

**Fig. 18:**
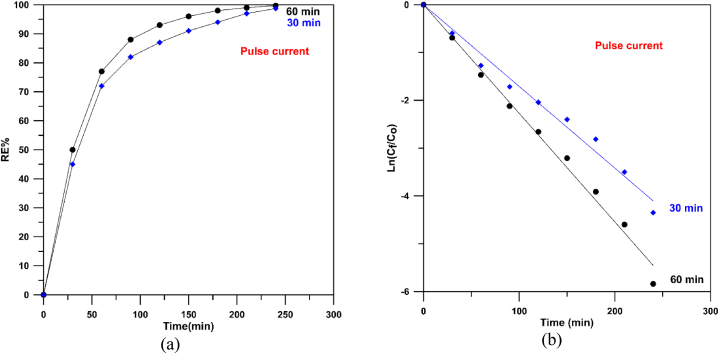
Decolorization of methylene blue at 250 rpm and 20 mA/cm^2^ (pulse) for 30 and 60 min. a) removal efficiency, b) Ln (C_f_/C_o_) versus time.

Fig. 18-a displays MB degradation by anodic oxidation utilizing different Cu/SnO_2_-Sb_2_O_5_ anodes prepared at 10 mA/cm^2^ (pulsed current) and 250 rpm for duration of 30 min and 60 min where the results displayed a good catalytic activity of the anode prepared at 60 min where removal efficiencies of 77 % and 99.7 % were achieved at 1 and 4 h respectively. Fig. 18-b shows the plot of ln (C_f_/C_o_) verses time for Cu/SnO_2_-Sb_2_O_5_ anodes prepared at 10 mA/cm^2^ (pulsed current) and 250 rpm for 30 and 60min. All fitting lines have R^2^ between 0.999 and 0.997 confirming the oxidation obeyed a pseudo first order behavior.

From Fig. 18-b, the rate constant at 60 min was found to be 0.0227 min^−1^ which is higher than that at 30 min (0.0179 min^−1^) by approximately 1.3 confirming the good performance and activity of Cu/SnO_2_-Sb_2_O_5_ anodes prepared by the cathodic deposition at 60 min. Additionally, the rate constant of present work is 3.8 times of that made by sol-gel method (0.006 min^−1^) [41] approving the good catalytic activity of Cu/SnO_2_-Sb_2_O_5_ prepared by cathodic deposition.

A comparison of the electrode activity based on the best conditions at Fig. 18-a in removal of methylene blue with similar SnO_2_ anodes is shown in Table 1. It was cleared that the new anode has good catalytic activity resulted from the combined effect of pulsing and rotation on the structure of anode which may increase the interior surface area and offer more active sites.

**Table 1:** The comparison of SnO_2_ activity with previous works.

Anode	mode	Method of preparation	Test conditions	Color removal (RE %)	Ref.
Ti/SnO_2_–Sb	Stationary with direct current	Electrodeposition	100 mg/L MB, 20 mA/cm^2^,4 h	89.6 %	32
Ti/SnO_2_–Sb	Stationary with direct current	Electrodeposition	100 mg/L MB, 20 mA/cm^2^,4 h	98.5 %	84
Ti/SnO_2_-Sb-CeO_2_	Stationary with pulsed current	Electrodeposition	100 mg/L phenol, 20 mA/cm^2^,4 h	90.7	44
Cu/SnO_2_-Sb_2_O_5_	Rotation with pulsed current	Electrodeposition	100 mg/L MB, 20 mA/cm^2^,4 h	99.7 %	Present work

### 3.2 Acceleration service life

**Fig. 19:**
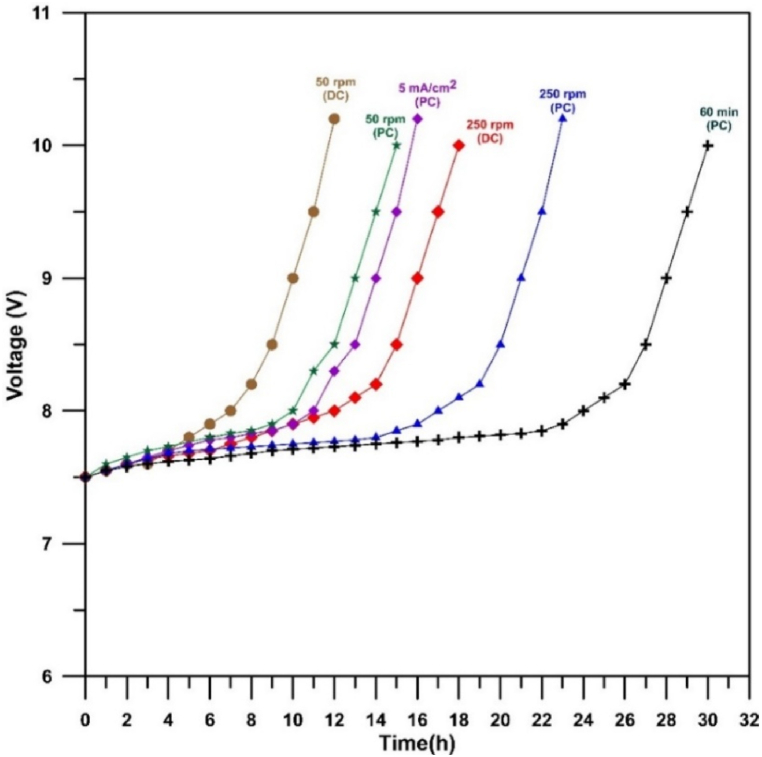
Accelerated service life curves for Cu/SnO_2_-Sb_2_O_5_ anodes.

Fig. 19 represents the accelerated service life of SnO_2_ anodes for the effect of rotation in two cases (direct and pulsed currents), effect of current (5 and 10 mA/cm^2^), and effect of time (30 and 60 min). It can be seen that increasing rotation gives higher service life time whatever the mode of current applied. Additionally at 250 rpm using pulsed current results in higher service life time in comparison with the direct current while longer time of deposition lead to increase the service life time of anode. In case of current density effect, indeed using 10 mA/cm^2^ gives better surface life time than 5 mA/cm^2^ but increasing the current density higher than 10 mA/cm^2^ results in lowering the activity of anode as shown in Fig. 15 and more porous deposits (Fig. 12-b). Hence it is not recommended to use current density higher than 10 mA/cm^2^. The maximum accelerated service life was 30 h in case of using 250 rpm, 10 mA/cm^2^ (pulsed current), and electrolysis time of 60 min. Furthermore, the actual service life time would be much greater than 30 h and depends on the conditions of oxidation process like pH, current density, and temperature [44].

(200 mA/cm^2^; 0.5 M NaOH).

Table 2 represents a comparison of the service life time for the present anode with those reported in the literature. For example, in comparison with Ti/SnO_2_–Sb_2_O_5_ prepared by thermal decomposition [65,84], the present work has higher service life. Additionally, the comparison with Ti/SnO_2_–Sb_2_O_5_ prepared by electrodeposition using stationary electrode with direct current supplying [85,86] showed that the present anode has higher acceleration service life. This is an indication on the formation a compact deposit on the anode surface prepared in this research as a result of rotation effect hence a high catalytic activity was obtained.

**Table 2:** The comparison of acceleration service life of Cu/SnO_2_-Sb_2_O_5_ with previous works.

Anode	mode	Method of preparation	Test condition	Service life time(h)	Ref.
Ti/SnO_2_–Sb_2_O_5_	Stationary	Thermal decomposition	0.5M NaOH; 200 mA/cm^2^	12	65
Ti/SnO_2_-Sb	Stationary	Thermal decomposition	1.0M NaOH; 500 mA/cm^2^	0.6	84
Ti/SnO_2_–Sb_2_O_5_	Stationary with direct current	Electrodeposition	0.5MH_2_SO_4_;100 mA/cm^2^	15	85
Ti/SnO_2_–Sb	Stationary with direct current	Electrodeposition	1.0M NaOH; 100 mA/cm^2^	12.1	86
Cu/SnO_2_-Sb_2_O_5_	Rotation with pulsed current	Electrodeposition	0.5MNaOH; 200 mA/cm^2^	30	Present work

Generally, the surface morphology plays an essential role on the stability of anode. During the test of service life, at high current density, the crystalline structure of anode would be altered irreversibly leading to deactivation of the anode. Moreover, generation of cracks during the electrolysis on the coating permits in penetrate of oxygen atoms formed by water electrolysis towered the surface of substrate resulting in formation an insulated layer of substrate oxide [41]. Besides, a passive hydration layer could be formed on the surface of anode results in fail of charge transfer and consequently increasing the voltage of cell [87].

Compared with boron doped diamond (BDD) and lead oxide anodes, BDD has better acceleration service life that can be achieved between 264 and 400 h at a current density of 1 A/cm^2^ in 3 M H_2_SO_4_ solution [88]. However, BDD is a high-cost anode and its preparation requires special equipment and procedures. As for lead oxide anode, the prepared anode in this work has a high acceleration service life. Kong et al. [89] found that Ti/PbO_2_ prepared by electrodeposition has an acceleration service life of 11 h at a current density of 1 A/cm^2^ in 3 M H_2_SO_4_ solution and improving this electrode with IrO_2_–Ta_2_O_5_ interlayer increased the acceleration service life to 672 h. However, lead ion contamination is the major drawback of these anodes.

### 3.3 Hospital wastewater treatment

The application of the prepared Cu/SnO_2_-Sb_2_O_5_ anode for treating wastewater generated from Al-Diwaniyah hospital by anodic oxidation was performed by varying the current density and pH during 120 min of degradation period.

#### 3.3.1 Effect of current density

**Fig. 20:**
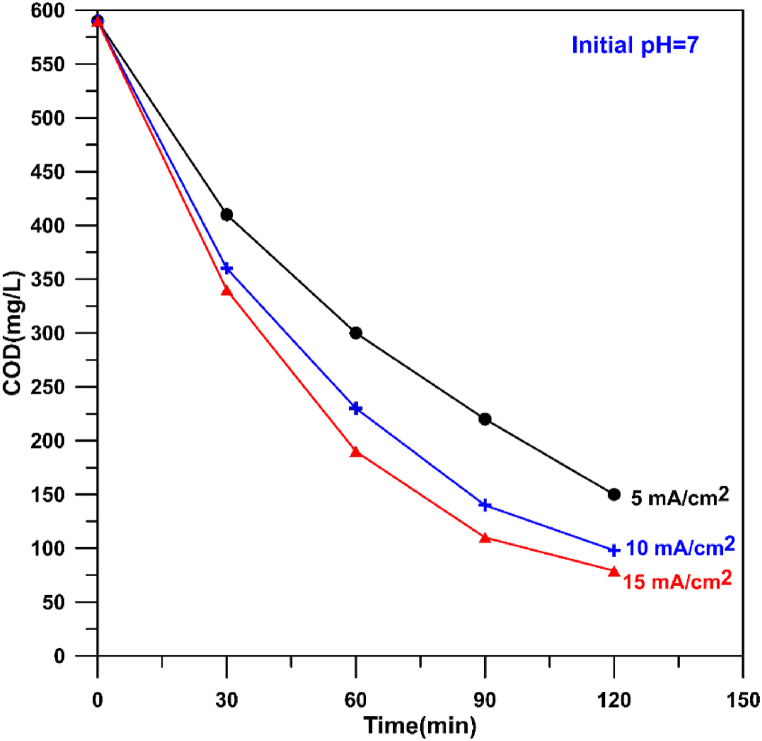
Influence of current density on the decay of COD during electro-oxidation of hospital wastewater using Cu/SnO_2_-Sb_2_O_5_ anode.

At the anodic oxidation, current density plays an effective role in degradation of organic pollutants because the rate of OH^•^ generation on the anode surface is proportional directly to the applied current [9]. Fig. 20 shows the effect of current density on COD removal at three values of current density (5, 10,15 mA/cm^2^) with an initial pH of 7. It was observed that an increase in current density results in a decrease in the final value of COD leading to removal efficiencies of 74.6, 83.4 and 86.6 % respectively. The corresponding energy consumption were 1.43, 3.03, and 6.13 kWh/kgCOD. Similar observation was noted by Wang et al. [9]in degrading ciprofloxacin using SnO_2_-Sb/Ti anode. A recent paper on degradation of ciprofloxacin using Ti/PbO_2_ anode confirmed the same behavior in spite of different anodic materials [25].

#### 3.3.2 Effect of pH

**Fig. 21:**
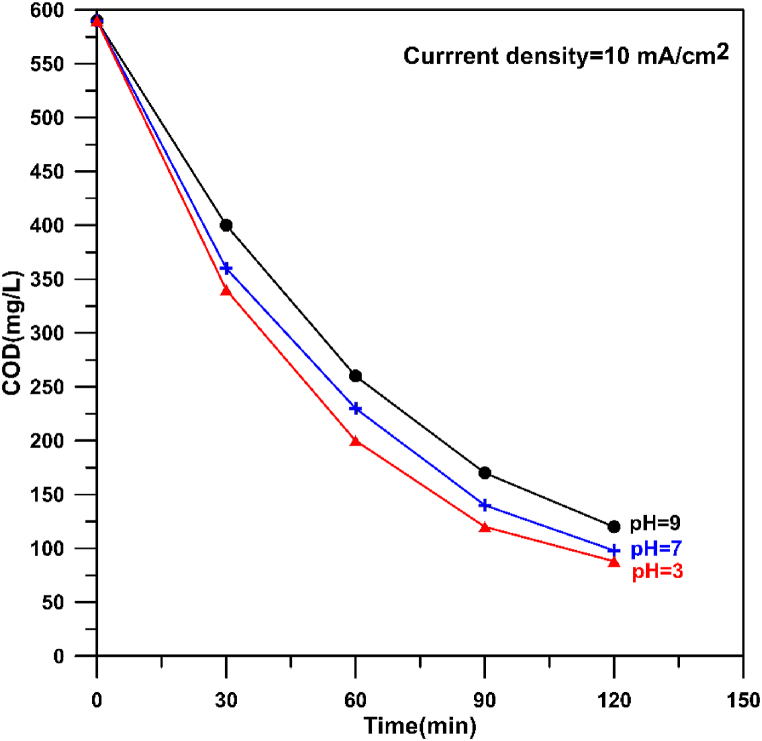
Influence of pH on the decay of COD during electro-oxidation of hospital wastewater using Cu/SnO_2_-Sb_2_O_5_ anode.

Fig. 21 shows the effect of initial pH on the COD removal at three values (3, 7, and 9) with a current density of 10 mA/cm^2^. It can be seen that increasing the pH from 3 to 9 results in increasing the final value of COD leading to removal efficiencies of 85, 83.4 and 79.66 % respectively. The corresponding specific energy consumption (SEC) were 2.85, 3.03, and 2.92 kW h/kgCOD respectively. The removal of COD at lower pH (3) is slightly higher than at pH of 7. This is because acidic conditions increase the oxygen overvoltage causing generation more OH• [90]. Furthermore, at alkaline conditions the conductivity of solution may be decreased due to the consumption of electrolyte leading to decrease the removal efficiency [91]. However, the overall difference in removal efficiency between pH of 3 and 9 is approximately 5 % which gives an indication on the possibility of application the prepared anode over a wide range of pH. The adverse effect of pH on COD removal was noted by previous works [14,25,26,92].

Furthermore, Phan et al. [93] showed that decreasing pH has a positive effect on the removal of COD (increasing COD removal efficiency) from pharmaceutical wastewater by anodic oxidation.

Zavala, et al. [94] found that acetaminophen is oxidized completely within 10 min at pH = 7, while at pH = 9, the time for complete oxidation of acetaminophen is 30 min confirming the good oxidation process at low pH.

Using boron doped diamond (BDD) anode in the oxidation of antihypertensive drug losartan, Dhawle et al. [95]found that the redox potential of hydroxyl radical is 2.8 V (vs. SHE) at pH lower than 3 while it was decreased to 1.6 V (vs.SHE) at pH greater than 9. This drop in redox potential causes lowering the removal rate of antihypertensive drug losartan at basic conditions.

#### 3.3.3 Comparison with previous work

Table 3 shows a comparison between the performance of present anode with those relevant SnO_2_ anodes used in treating hospital wastewaters. The prepared anode has the ability to reduce COD from 590 mg/L to 88.5 mg/L achieving COD removal of 85 % with 120 min while previous works required longer time with starting at low concentrations. The treatment effluent has BOD of 34 mg/L below the standard limit for discharging hospital wastewater.

**Table 3:** Comparison the performance of present anode with relevant anodes in previous works.

Wastewater	Anode	Conditions	Removal efficiency	Reference
ciprofloxacin	Ti/SnO_2_-Sb	CIP(50 mg/L),pH = 5.4, 30 mA/cm^2^; T = 30 °C,0.05M Na_2_SO_4_,120 min,	COD = 86.0 % 11.61 Wh/L	[27]
ciprofloxacin	Sb-doped SnO2	CIP(30 mg/L),pH = 3, 20 mA/cm^2^; T = 30 °C,0.176M Na_2_SO_4_, 81.87 %	100 %	[96]
ciprofloxacin	Ti/BDD	CIP(50 mg/L),pH = 3, 30 mA/cm^2^; T = 30 °C,0.05M Na_2_SO_4_,90 min,	COD = 92.4 % 35 W h dm^−3^.	[26]
ciprofloxacin	PbO_2_	CIP(15 mg/L),pH = 3, 16 mA/cm^2^; T = 30 °C,0.176M Na_2_SO_4_, 81.87 %, 75 min	COD = 88.8 %% 15.77649 kW h/m^3^	[25]
Ceftazidime (CFD)	Ti/TiO_2_/SnO_2_-Sb-Cu	5 mg L −1 pH = 2, 1.25 mA/cm^2^; T = 30 °C,0.007M Na_2_SO_4_, 81.87 %	81.87 %	[97]
pharmaceutical wastewater	Ti/RuO_2_-IrO_2_-SnO_2_	8 mA/cm^2^, initial pH = 2, 24 h	95.92 % 58.09 kWh/kgCOD	[98]
pharmaceutical wastewater	Cu/SnO_2_-Sb_2_O_5_	COD = 590 mg/L 10 mA/cm^2^ 120 min, pH = 3	85 % 2.85 kWh/kgCOD	Present work

The energy consumption in the present work is relatively lower than previous works that used Ti/SnO_2_–Sb_2_O_5_ anode in degrading various types of pharmaceutical wastewaters [27,96] in spite of using wastewater with high initial value of COD in the present work.

Table 3 shows that BDD has higher ability than PbO_2_ in degrading ciprofloxacin with high concentration. However, the proposed anode has the ability to degrade more complex wastewater at good efficiency and energy consumption.

There are several potential challenges in scaling up anodic oxidation using a rotating cylindrical anode for real-world applications. In this field, more pilot –scale researches at larger treatment capacity are needed to test the feasibility of anodic oxidation for real –world application. Additionally, industrial application of anodic oxidation needs validation the performance at larger scale. On the other hand scale-up of electrooxidation reactors requires thoughtful assessment of hydrodynamic, design of anode, and reactor dimensions. Cleaning of anode and cathode in the electrochemical reactor should be considered in scale-up by adopting feasible approaches to perform long-term operation for the reactor especially for wastewaters containing high organic pollutants and high salinity such as hospital wastewaters. The economic analysis of anodic oxidation confirmed that anode material constitutes the main cost of the whole system. Therefore, utilizing anodes have high efficiency with low cost of material and long service life time would be a challenge in scale-up of anodic oxidation processes [99]. Moreover, the scalability and adaptability of anodic oxidation to treat different wastewater compositions need further attention. The activity of anodic oxidation depends on the specific wastewater properties, such as pH, conductivity, and organic load. Therefore, the optimization process among these variables is essential to ensure efficient and reliable operation [100].

Wastewater contains a variety of inorganic and organic contaminants, including organic compounds, heavy metals, and pharmaceuticals. Therefore, the simultaneous treatment of multiple pollutants with different chemical properties would be a challenge. The presence of one pollutant may interfere with degrading or removing the others, affecting the overall treatment efficiency. The interactions between different pollutants and their effects on the mechanism of anodic oxidation deserve careful study [101]. To address these challenges and limitations, future research efforts should be directed to understand anodic oxidation in field-scale applications while discovering innovative electrode materials and designs that can increase electrode durability and reducing the pollution.

## 4 Conclusion

A new approach composed of applying rotation with pulsed current in preparation of Cu/SnO_2_-Sb_2_O_5_ anode by cathodic deposition has been applied successfully in which high active with low cost SnO_2_ can be prepared and scale up industrially. Results showed that application of cathodic deposition with using direct current at a rotation of 250 rpm gives better properties than 50 rpm in terms of compact deposits, high activity toward MB degradation, and longer service life time. Application pulsed effect of current results in enhancement the properties of Cu/SnO_2_-Sb_2_O_5_ anode. Increasing the current density up to 10 mA/cm^2^ would enhance the properties of anode in comparison with 5 mA/cm^2^. However, higher current density gave the adverse effect of needle like deposits and low activity toward MB degradation. Additionally using longer period of cathodic deposition gives excellent Cu/SnO_2_-Sb_2_O_5_ anode with accelerated life time reach to 30 h. Application of the prepared anode in treating hospital wastewater gave an excellent removal of COD (85 %) with COD final of 88.5 mg/L (lower than the discharged limits for environment) when operating at a current density of 10 mA/cm^2^ and period of electrolysis equal 2 h. In this case, energy consumption of 2.85 kWh/kgCOD was required which is relatively low confirming the high activity of the prepared Cu/SnO_2_-Sb_2_O_5_ anode owing to the simultaneous impact of rotation and pulsing.

The results of this work provide a strong incentive and encourage research in the field of wastewater treatment by anodic oxidation and commercial purposes for moving to a new level of innovation based on the low-cost quality concept presented here. The prepared Cu/SnO_2_-Sb_2_O_5_ anode can be scaled up and used to treat various organic wastewater to examine the feasibility of its potential scale-up application. Adopting rotating electrode design would be made the scale-up to industrial applications more feasible. Doping the new anode with other elements or using interlayer between SnO_2_ and copper substrate could be improve the stability of anode. A research in this direction would be a promising step.

## CRediT authorship contribution statement

**Falah H. Abd:** Writing – original draft, Validation, Project administration, Methodology, Data curation, Conceptualization. **Ali H. Abbar:** Writing – review & editing, Writing – original draft, Visualization, Validation, Supervision, Software, Resources, Project administration, Methodology, Investigation, Formal analysis, Data curation, Conceptualization.

## Data availability statement

Data will be made available on request. For requesting data, please write to the corresponding author.

## Declaration of competing interest

The authors declare that they have no known competing financial interests or personal relationships that could have appeared to influence the work reported in this paper.
